# Enhancing cybersecurity: A high-performance intrusion detection approach through boosting minority class recognition

**DOI:** 10.1371/journal.pone.0317346

**Published:** 2025-03-28

**Authors:** Chadia E. L. Asry, Ibtissam Benchaji, Samira Douzi, Bouabid E. L. Ouahidi

**Affiliations:** 1 IPSS Laboratory, Faculty of Sciences, Mohammed V University, Rabat, Morocco; 2 Faculty of Medicine and Pharmacy, Mohammed V University, Rabat, Morocco; Cardiff Metropolitan University - Llandaff Campus: Cardiff Metropolitan University, UNITED KINGDOM OF GREAT BRITAIN AND NORTHERN IRELAND

## Abstract

The swift proliferation and extensive incorporation of the Internet into worldwide networks have rendered the utilization of Intrusion Detection Systems (IDS) essential for preserving network security. Nonetheless, Intrusion Detection Systems have considerable difficulties, especially in precisely identifying attacks from minority classes. Current methodologies in the literature predominantly adhere to one of two strategies: either disregarding minority classes or use resampling techniques to equilibrate class distributions. Nonetheless, these methods may constrain overall system efficacy. This research utilizes Shapley Additive Explanations (SHAP) for feature selection with Recursive Feature Elimination with Cross-Validation (RFECV), employing XGBoost as the classifier. The model attained precision, recall, and F1-scores of 0.8095, 0.8293, and 0.8193, respectively, signifying improved identification of minority class attacks, namely “worms,” within the UNSW NB15 dataset. To enhance the validation of the proposed approach, we utilized the CICIDS2019 and CICIoT2023 datasets, with findings affirming its efficacy in detecting and classifying minority class attacks.

## 1. Introduction

Progress in network communication technologies, the Internet of Things (IoT), and cloud computing has become essential to contemporary civilization [1–3]. These advancements have exacerbated the intricacies of cybersecurity, since cyber threats now present substantial risks to individuals, organizations, and governments together [4].

In 2023, Imperva’s “2023 Bad Bot Report” indicated that malicious bots, software engineered for detrimental automated functions, constituted 32% of global online traffic, rising from 30.2% in 2022 [5].

Malicious intrusions represent a considerable segment of network security incidents, presenting major threats to both persons and companies. Consequently, Intrusion Detection Systems (IDS) are essential for safeguarding hosts and systems [6]. Intrusion Detection Systems (IDS) constitute an essential element of security infrastructure, designed to thwart cyber threats from diverse categories of attackers. Security literature categorizes IDS into two primary types: host-based IDS [7], which observes events and traffic on specific computers, and network-based IDS, which safeguards the entire network [8]. Network IDS approaches are classified into flow-based methods, which examine packet header information, and deep packet inspection methods, which offer a more comprehensive analysis. Intrusion Detection System (IDS) methodologies can be categorized according to their detection strategies, which encompass signature-based detection and anomaly-based detection techniques.

Host IDS focuses on securing a computer system by monitoring events and traffic, whereas network IDS secures the entire computer network. Network-based IDS techniques are divided into flow-based solutions [9] and deep packet inspection schemes [10]. Flow-based methods analyze only the packets’ header information, while deep inspection techniques offer a more comprehensive examination. IDS techniques can be categorized by their detection capability into signature detection and anomaly detection approaches [11].

Signature-based Intrusion Detection Systems (IDS) utilize a predefined database of security attack signatures to identify and correlate events and network traffic with known attack patterns [12]. These IDS methods are inefficient at identifying novel attacks with unknown patterns and signatures. Anomaly-based Intrusion Detection System (IDS) techniques focus on identifying abnormal actions by learning typical patterns and identifying deviations as anomalies or intrusions [13]. However, they encounter the problem of false positives, which restricts their practical use. Despite attempts to enhance network security, the rising quantity of novel threats and network intricacy emphasize the necessity of establishing dependable security procedures to oversee, scrutinize, and safeguard network systems’ functions [14]. Diverse solutions are needed to create and execute more effective intrusion detection systems. Statistical and machine learning methods have been used to forecast network intrusions [15].

Deep learning and machine learning techniques are crucial in network security detection, utilizing large datasets to properly forecast and address possible attacks. These datasets in network security contain records of normal network traffic and different types of network intrusions. However, a major challenge is the imbalance in these datasets, with normal traffic instances significantly outnumbering instances of network intrusions. This skewed distribution impacts the learning process of these models.

The imbalance significantly affects the effectiveness of intrusion detection systems (IDS) as they often show a bias towards the majority class, which is regular traffic, due to encountering it more frequently during training. Consequently, these models excel at recognizing typical behavior but struggle to detect uncommon, minority attacks. This biased portrayal results in a lower detection rate for such assaults, despite their ability to cause significant harm to network systems.

Worm attacks vividly demonstrate the risks linked to these minority categories. Worms are malevolent software programs created to propagate via networks by taking advantage of weaknesses in software or network setups. Worms may self-replicate and spread autonomously, unlike other forms of malware, rapidly moving from one machine to another. Worms can rapidly reproduce and use up significant network and system resources due to their capacity to replicate autonomously. Worms spreading quickly can severely damage network infrastructure, cause extensive downtime, and lead to essential data and services being lost. Worms can carry out various damaging operations such as destroying data, stealing information, installing backdoors, and interrupting network functions. Worm assaults are especially dangerous due to their quick dissemination, ability to self-replicate, and capacity to carry harmful payloads.

In order to tackle the significant problem of class imbalance in network security datasets, specifically for underrepresented classes like Worm, R2L (Remote to Local), and U2R (User to Root) assaults, researchers have put up a range of creative alternatives. The objective of these solutions is to improve the detection capabilities of Intrusion Detection Systems (IDS) for these less often recognized but very harmful forms of attacks.

Liu et al. [16] introduced a new approach called Difficult Set Sampling Technique (DSSTE) to address the issue of class imbalance in datasets such as NSL-KDD and CSE-CIC-IDS2018. The algorithm generates synthetic samples for minority classes, improving the performance of classifiers. Bedi et al. [17] presented Siam-IDS, an Intrusion Detection System (IDS) based on Siamese Neural Network. The main objective of Siam-IDS is to tackle the issue of class imbalance, without relying on conventional balancing methods such as oversampling and undersampling. Their methodology yielded superior recall values for the R2L and U2R attack categories, demonstrating the promise of deep learning in addressing class.Another interesting advancement involves the utilization of Generative Adversarial Networks (GANs) for the purpose of enhancing data in underrepresented categories. Alabrah [18] proposed the utilization of a GAN model to produce artificial examples of underrepresented categories, hence improving the effectiveness of machine learning classifiers on the UNSW-NB15 dataset. The method has demonstrated encouraging outcomes in enhancing the levels of accuracy, precision, and F1-scores when it comes to detecting attacks on minority groups. Elghalhoud et al. [19] introduced a Convolutional Neural Network (CNN) based Network Intrusion Detection (NID) system that specifically tackles the issue of imbalanced datasets and successfully categorizes various types of attacks.

Kasongo [20] built an IDS that incorporated numerous machine learning algorithms, such as Linear Regression (LR), Random Forest (RF), Naive Bayes (NB), Decision Tree (DT), Extra Trees (ET), and Extreme Gradient Boosting. Furthermore, they used a Genetic Algorithm (GA) to select features and incorporated RF as the fitness function for the GA. Using the UNSW-NB15 dataset, they evaluated the model’s performance. Consequently, the model obtained an Area Under the Curve (AUC) value of 0.98 and an accuracy of 87.61%. Gao et al. [21] introduced an ensemble machine learning intrusion detection system (IDS) that employs the Principal Component Analysis (PCA) method for feature extraction in their research. Their methodology entails the integration of numerous classifiers, such as Decision Tree, Random Forest, K-Nearest Neighbor (KNN), Deep Neural Network (DNN), and MultiTree, through a majority vote ensemble algorithm that allocates weights to each classifier in order to enhance the overall accuracy. Their experimental evaluation on the NSL-KDD dataset resulted in an accuracy of 85.2%, surpassing the accuracy of any individual classifier when used independently. Nevertheless, the model’s efficacy is compromised when it is used to analyze rare attacks that occur infrequently. Kasongo et al. [22] conducted a study in which they examined a novel intrusion detection system that employs a feed-forward deep neural network (FFDNN) and a wrapper-based feature extraction unit (WFEU). The results indicated that the system performed well in binary classification; however, it was subpar in multi-classification scenarios. The authors of [23] conducted a comparison of the performance of a variety of classifiers, such as the Decision Tree, Simple Logistic Regression, Naïve Bayes, Multi-layer Perceptron, Support Vector Machine, Random Forest, and Zero Rule, and determined that the Decision Tree classifier was the most effective in detecting intrusions. Pham et al. [24] proposed an IDS framework that employed the gain ratio feature selection technique in conjunction with an ensemble bagging model, with J48 serving as the base classifier. The bias toward features with a large number of values was surmounted by the J48 algorithm in this study through the use of gain ratio feature selection. Their methodology has been contrasted with the ensemble of K-Nearest Neighbors (KNN) and correlation feature selection with Naive Bayes. The experimental results indicated that the proposed framework enhanced classification accuracy and reduced the rate of false alarms. Devikrishna et al [25] implemented the Multi-Layer Perceptron (MLP) architecture to classify assaults into six categories and detect intrusions. Nevertheless, the MLP approach was regarded unsuitable as a result of its irrelevant output. The authors of [26] propose a novel method for intrusion detection that entails the partitioning of network data into smaller subsets using a C4.5 decision tree algorithm, followed by the development of multiple SVM models for each subset. This approach effectively decreases the complexity of time and improves the rate of detecting unknown attacks. Maseer et al. [27] developed a hybrid deep learning-based IDS for IoT that employs a weighted Deep Belief Network (DBN). A Gaussian-Bernoulli model is included in the model. A weighted Deep Neural Network and a Restricted Boltzmann Machine. The CICIDS2017 dataset was employed by the researchers to assess the model’s performance. Consequently, the model obtained a 99.38% accuracy rate for web attacks and a 99.99% accuracy rate for both attacks. Guezzaz et al. [28] improved the quality of data on both the NSL-KDD and CIC-IDS2017 datasets by developing an IDS model that employed the Decision Tree (DT) algorithm. Subsequently, the researchers conducted a comparison between the model’s outcomes and those of comparable models that utilized the same datasets. Their results indicated that the proposed model obtained an accuracy rate of 99.42% and 98.8% for the NSL-KDD and CIC-IDS2017 datasets, respectively.

The aforementioned research makes substantial contributions to the field of Network Intrusion Detection Systems (NIDS) by employing diverse machine learning techniques and feature selection approaches. Nevertheless, there are many constraints in their methodologies that could impede the overall efficiency of intrusion detection, particularly in the detection of specific types of attacks and the resolution of the challenge posed by minority class attacks.

Firstly, the approach of picking pertinent variables from the complete dataset, as employed in these studies, may not be the most effective method for classifying specific types of attacks. This approach is predicated on the notion that the characteristics that are most relevant to the entire dataset are also the most relevant for identifying each specific type of attack. However, certain assaults may exhibit unique patterns or behaviors that require specific sets of criteria for accurate detection. The intrusion detection process may be susceptible to potential weaknesses if significant features that are essential for detecting specific assaults are disregarded due to a failure to customize feature selection for each attack type.

In addition, most of these researches place accuracy as the foremost criterion for assessing their models. Although accuracy is undeniably a crucial measure, it alone does not offer a comprehensive assessment of a model’s effectiveness in intrusion detection scenarios. Within the framework of NIDS, the parameter of recall (also known as the true positive rate) has significant importance. Recall quantifies the ratio of accurately diagnosed system attacks to the total number of real attacks. High recall is crucial in intrusion detection as the failure to detect real attacks (false negatives) might result in more severe effects than mistakenly classifying regular traffic as assaults (false positives). By prioritizing accuracy, these studies may unintentionally downplay the significance of reducing missed attacks, which is a crucial element of an efficient NIDS.

Furthermore, the failure to consider attacks targeting minority groups in this research is a notable omission. Intrusion detection datasets frequently demonstrate class imbalance, wherein the number of occurrences of assaults (particularly specific types of attacks) is much lower compared to normal traffic. The disparity in class distribution is a difficulty for machine learning models, as they may exhibit bias towards the dominant class (regular traffic) and exhibit subpar performance on the less prevalent classes (special attacks). If this mismatch is not properly addressed, the models run the danger of failing to detect infrequent but possibly catastrophic threats. This overlook highlights the necessity for strategies that not only prioritize general correctness but also guarantee the efficient identification of assaults targeting minority classes. This is crucial for establishing strong and all-encompassing network security.

This article introduces an approach to address the problem of imbalanced data in network intrusion detection systems, particularly for the identification of minority classes such as worm, R2L, and U2R attacks. Our technique obviates the need for data augmentation. We want to optimize the usefulness of the current data set by employing advanced techniques for feature selection and classification.

This approach is bolstered by additional noteworthy contributions that are focused on enhancing the efficiency and accuracy of our system:

1. We present a new approach for dividing datasets using the frequency of attacks, utilizing the ShapRFECV algorithm. This approach integrates SHAP (SHapley Additive exPlanations) with RFECV (Recursive Feature Elimination with Cross-Validation) to precisely detect and preserve the most influential features for each type of assault, hence guaranteeing a targeted and effective feature set.
2. Our approach incorporates a distinctive Feature Frequency Combination technique, which combines crucial features to enhance their overall predictive capability. Reinforcing the detection capabilities of our model is essential, especially for minority attack types.
3. We customize our model to properly handle imbalanced datasets and enhance classification metrics such as Accuracy, Precision, Recall, and F1-score. This emphasis is crucial for improving the identification efficiency of underrepresented categories without depending on data augmentation.
4. We conducted a thorough evaluation of the effectiveness of our proposed model using the UNSW-NB15 dataset. The experimental findings exhibited significant enhancements in classification performance across crucial parameters, confirming the usefulness of our approach in tackling imbalanced data issues.

These contributions represent a thorough and efficient approach to identifying significant security vulnerabilities caused by minority groups in network intrusion detection systems. They establish a new standard for addressing imbalanced data without the requirement of expanding the dataset.

The ensuing sections of the paper are arranged in the following manner: The Background section offers a comprehensive explanation of the model utilized in the present study. The Methodology, Experiment, and Result Analysis section provides a thorough elucidation of the suggestedapproach and meticulously scrutinizes the experimental data. The conclusion section offers a succinct summary of the main findings provided in the study.

## 2. Materials and methods

### 2.1. SHAP feature importance

SHAP (SHapley Additive exPlanations) [29] is a technique employed to elucidate the outcome of machine learning models by calculating the individual impact of each feature on the prediction. The significance of a certain attribute, as assessed by SHAP, is calculated by taking the average of the absolute Shapley values generated for a specific dataset. Shapley values, which are developed from cooperative game theory, quantify the individual impact of each feature on the prediction by considering all potential combinations of features.

In order to assess the significance of each feature, the focus is directed towards the absolute Shapley values. Features with high absolute Shapley values are deemed relevant as they exert a greater influence on the model’s predictions. The procedure encompasses the subsequent stages:

- Calculate the Shapley values: Determine the Shapley value for each feature in every data point. This value represents the extent to which the feature affects the discrepancy between the actual forecast and the average prediction.
- To determine the overall significance of a feature, we compute the mean of the absolute Shapley values for that feature throughout the whole dataset. The formula for this is as follows:

$$\text{Fn}=\frac{1}{N}\sum_{i=1}^{N}\left|\text{φ}\begin{array}{c} \left(i\right)\\ n \end{array}\right|$$ (1)

Where Fn represents the average Shapley value for the nth feature, N represents the total number of samples in the dataset and $\text{φ}\begin{array}{c} \left(i\right)\\ n \end{array}$ denotes the Shapley value for the nth feature in the ith data point:


$${\text{φ}}_{n}\left(i\right)=\underset{S\subseteq N\left\{n\right\}}{\sum }\frac{\left|S\right|!\left(\left|N\right|-\left|S\right|-1\right)!}{\left|N\right|!}[f\left(S\cup \left\{n\right\}-f\left(S\right)\right]$$

Where:

S is a subset of features excluding feature n.

|S| is the size of the subset S.

f(S) is the model prediction using the subset of features S.

The term $\frac{\left|S\right|!\left(\left|N\right|-\left|S\right|-1\right)!}{\left|N\right|!}$is a coefficient that guarantees the consideration of all potential arrangements of the features.

**f(S)** represents the prediction generated by the model using only the features included in the subset S.

**f(S**∪**{n}**) represents the prediction generated by the model when the characteristics in the subset S, in addition to the characteristic n, are utilized. It denotes the result of the model when the supplementary information from feature n is incorporated.

The quantity **f(S**∪**{n}) − f(S)** represents the alteration in the forecast caused by the addition of feature n. This modification represents the incremental impact of feature n on the model’s prediction when it is included in the subset S of features.

### 2.2. Recursive feature elimination with cross-validation (RFECV)

Recursive feature elimination (RFE) is a feature selection technique that improves the performance of a model by methodically eliminating redundant and weak features, hence reducing their influence on training error. RFE utilizes a method called backward feature elimination [30], which involves an iterative process.

Consider a set of features, X, where X = {X1, X2,..., Xn}, and n represents the total number of features. In the training process, we utilize all the features in the dataset to train the model. The Recursive Feature Elimination (REF) ranks the features according to their importance scores. The scores can be obtained from several metrics, depending on the model employed, such as coefficients in linear models or feature importances in tree-based models. Let I =  {I_1_, I_2_,..., I_n_} represent the importance scores associated with characteristics X. Iterative elimination removes the least important feature, denoted as *Xmin*, by using recursive feature elimination (REF). The model is then retrained on the reduced set of features, ***X*′* = X*∖{*Xmin*},** and the importance scores for the remaining features are recalculated. This process is repeated until the predefined number of features, k, is reached.

### 2.3. UNSW NB15 dataset

The UNSW-NB15 dataset, introduced in 2015, encompasses a diverse range of modern network vulnerabilities, encompassing nine distinct types of cyber-attacks. This dataset contains a complete collection of 45 attributes, encompassing 221,876 instances of normal interaction and 321,283 instances of assault events. It serves as a crucial standard for evaluating the effectiveness of network intrusion detection systems. The characteristics are classified into six clearly defined groups: Core Features, Flow Features, Time Features, Content Features, Additional Generated Features, and Labeled Features. Features 36 to 40 are categorized as general-purpose, while features 41 to 45 are specific to connections. The dataset comprises nine unique attack categories (Table 1): Analysis, Fuzzers, Backdoors, DoS, Exploits, Reconnaissance, Generic, Shellcode, and Worms. Every category has distinct challenges for cybersecurity defenses [31,32].

**Table 1:** Distribution of training and testing data by connection type from the UNSW-NB15 dataset [25].

Type connection	Train	Test
Normal	56000	37000
Analysis	2000	677
Backdoor	1746	583
DoS	12264	4089
Exploits	33393	11132
Fuzzers	18184	6062
Generic	40000	18871
Reconnaissance	10491	3496
Shellcode	1133	378
Worms	130	44

- Fuzzers: This attack technique involves overwhelming a system with significant amounts of random data to uncover flaws that could potentially cause the system to crash. The goal is to capitalize on vulnerabilities in software, operating systems, or networks.
- Scanning is an early type of intrusion that involves techniques like port scanning and the dissemination of spam or HTML files. The objective of this is to identify weaknesses that can be manipulated to gain entry into online applications.
- Backdoors are clandestine strategies employed by assailants to circumvent standard authentication protocols, enabling them to attain illicit remote entry to a system while endeavoring to evade detection.
- DoS, short for Denial of Service, is an intentional attack that seeks to disrupt a system’s resources by inundating its memory with an excessive number of requests. This results in legitimate users being unable to use the system.
- Exploits involve executing code to take advantage of vulnerabilities, weaknesses, or unintended behaviors in host systems or networks with the aim of gaining unauthorized access or control.
- Generic refers to a strategy that applies collisional hash functions to all cipher blocks, regardless of their configuration.
- Reconnaissance is a tactic used in an assault to collect information by thoroughly studying computer systems and networks, with the goal of overcoming network security limitations.
- Shellcode is a technique employed by attackers to gain unauthorized access to a system by injecting a small piece of code or a shell, enabling them to assume control over the compromised machine.
- Worms are a form of malicious software that may replicate and spread over computer networks. They frequently use vulnerabilities in the network to proliferate and obtain illegal entry into computers.

**Fig 1:**
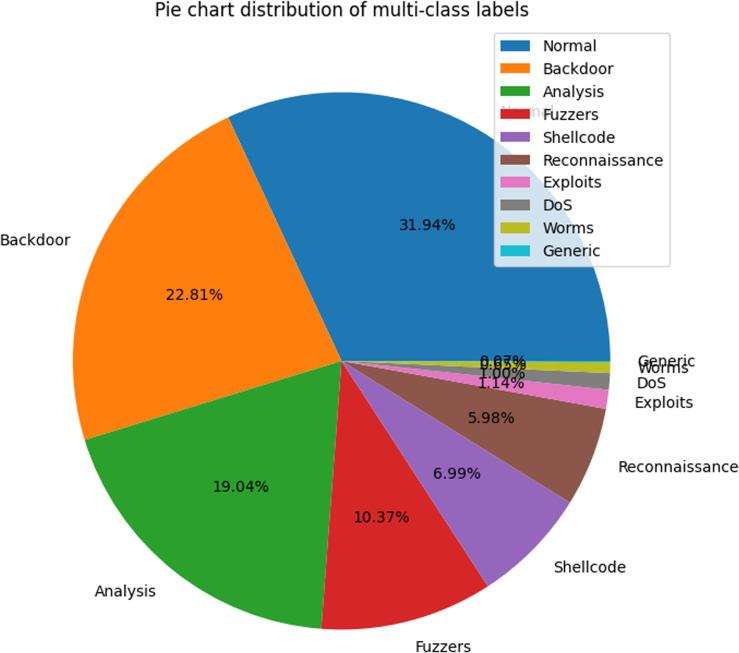
Distribution of multi-class labels in the UNSW-NB15 dataset.

The unequal distribution of various attack categories in the UNSW-NB15 dataset is illustrated by the pie chart in Fig 1. This graphic emphasizes a substantial imbalance, as normal records account for more than one-third (31.94%) of the data. In striking contrast, the data pertaining to worm attacks constitutes only a negligible portion (0.007%) of the total. The efficacy and accuracy of classification algorithms can be influenced by such a severe class imbalance, which may result in biased model predictions [33].

The UNSW-NB15 dataset offers a plethora of advantages for network intrusion detection research [34]. It is relevant to current cybersecurity challenges due to its combination of synthetically generated contemporary attack activities and actual network events. Furthermore, the dataset encompasses a wide range of data that was gathered from both the payload and packet metadata, which enables a thorough examination of network traffic.

Nevertheless, the UNSW-NB15 dataset’s intricate nature is indicative of the subtlety and complexity of contemporary attack patterns. This complexity presents a challenge for researchers who are striving to create algorithms that are capable of identifying emergent threats. This task is further complicated by the underrepresentation of specific types of attacks, including User-to-Root (U2R), Remote-to-Local (R2L), and Worms. The enhancement of detection algorithms for these attack types that are inadequately represented is a critical area of focus for the advancement of network security.

## 3. Proposed approach and experimental results

**Fig 2:**
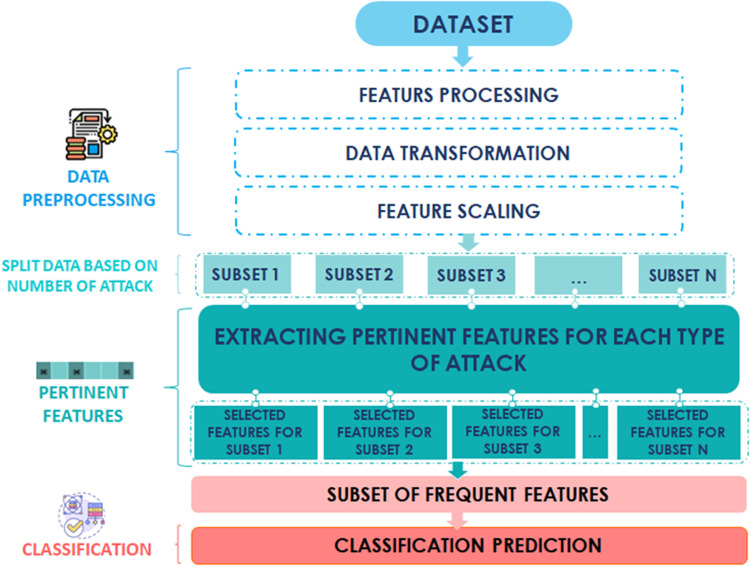
Flow chart of the proposed approach.

The proposed methodology, illustrated in Fig 2, comprises four sequential steps: data preprocessing, data splitting, feature selection, and classification. The next sections provide a comprehensive explanation of these steps.

### 3.1. Data preprocessing

Traffic data often contains noisy, redundant, and varied types of data, which presents significant challenges for knowledge discovery and data modeling. Therefore, data preparation is crucial to guarantee the usability of the data for the advancement of any learning algorithm. In this study, the procedure involves the following steps:

- Feature removal: The UNSW-NB15 dataset comprises 45 attributes and two labels. The ‘id’ has been eliminated due to its insignificant influence on the classification of network traffic.
- Transforming categorical data into numerical variables.
- Data normalization is the procedure of standardizing the values of data features to ensure they are on a comparable scale. This is done to prevent a model from mistakenly seeing features with bigger values as having a more significant influence on the outcomes. The Min-Max normalization method has been selected for this experiment. Consequently, each characteristic is adjusted to fit within the numerical interval of 0 to 1, utilizing the subsequent mathematical formula:

$$X_{n}=\frac{x-x_{\min}}{x_{\max}-x_{\min}}$$

Where x_n_ represents the normalized data, x is the original value to be processed, and x_max_ and x_min_ indicate the maximum and minimum data values in the current attribute, respectively.

Upon completion of the data processing, the dataset is partitioned into nine subsets according to the form of attack (see Table 2). Subset 1, for instance, comprises instances of the generic attack type.

**Table 2:** Partitioning the dataset into a set of subsets.

Subset	Attacks	Number of instances
Subset 1	Generic	40000
Subset 2	Exploits	33393
Subset 3	Fuzzers	18184
Subset 4	DoS	12264
Subset5	Reconnaissance	10491
Subset 6	Analysis	2000
Subset 7	Backdoor	1746
Subset 8	Shellcode	1133
Subset 9	Worms	130

### 3.2. Feature selection for each attack using SHAP-based recursive feature elimination (RFE)

In the fields of machine learning and data science, the process of creating predictive models that are both accurate and efficient requires the careful selection of the most suitable set of attributes. Having too many features can result in overfitting, higher computing costs, and worse model performance. The objective is to determine the most influential characteristics that improve the model’s capacity to make accurate predictions while eliminating superfluous components [35].

Incorporating the game-theoretical methodology known as SHAP (SHapley Additive exPlanations) into the standard RFE method allows for its enhancement. Recursive Feature Elimination with Cross-Validation (RFECV) and SHAP are utilized to assess and extract the optimal set of features based on SHAP importance scores. The Light Gradient Boosting Machine (LGBM) model is used as the primary classifier in this approach. The procedure for each iteration of RFECV-SHAP is as follows:

- Optimizing model parameters through the use of Randomized Search CV for fine-tuning model hyperparameters.
- Utilizing cross-validation to train the model on the training data and assess its performance on validation data using the Area Under the Curve (AUC) measure.
- Eliminating the k features with the lowest SHAP significance from the dataset.
- The performance of the suggested RFECV-SHAP feature significance technique utilizing the LGBM classifier is
- Visualized by plotting the AUC.

This strategy guarantees that the chosen features provide a substantial contribution to the predictive ability of the model, while also dealing with the difficulties of adjusting model hyperparameters and reducing the bias towards features with a high number of distinct values in the standard Recursive Feature Elimination (RFE) method.

**Fig 3:**
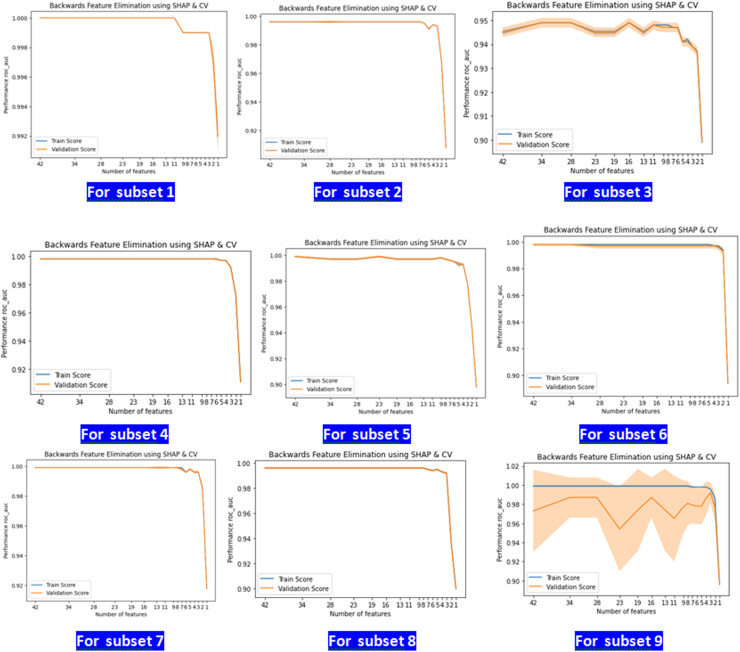
Results of SHAP-based recursive feature elimination with cross-validation (RFECV) applied to the LightGBM (LGB) model for each subset.

The exhibited figures (Fig 3.) depict the performance of the Recursive Feature Elimination with Cross-Validation utilizing SHAP (RFECV-SHAP) on nine distinct subsets. The performance is evaluated by calculating the Area Under the Curve (AUC) metric, which is then plotted against the number of features for both the training and validation sets.

Each subset displays an optimal range of features in which the AUC performance reaches its highest point. The range of values varies slightly among different subsets, highlighting the importance of selecting features relevant to each subset in order to obtain optimal model performance.

**Subset 1 and Subset 2:** These subsets exhibit a notably high initial AUC that remains consistent until there is a significant reduction in the number of features, at which point the validation score significantly declines. This indicates that a few extremely important characteristics have a strong influence on the performance of the model.

**Subset 3:** Exhibits a progressive decrease in validation score, accompanied by notable swings. The variability observed indicates that the subset may contain characteristics that have different levels of significance and potential interactions that impact the stability of the model.

**Subset 4, 5, 6, and 7:** These subsets exhibit a trend akin to subsets 1 and 2, with consistent AUC values until a critical number of characteristics are eliminated, suggesting the existence of essential features that greatly impact model performance.

**Subset 8:** Has a consistent decrease in validation performance as features are removed, indicating a gradual dispersion of feature relevance without a distinct set of dominant characteristics.

**Subset 9:** exhibits notable variations in validation AUC, suggesting the presence of a potentially intricate feature set with interactions that have a non-linear impact on the model.

The findings from the RFECV-SHAP analysis emphasize the significance of customizing feature selection for each subset to improve the performance of the model. The existence of crucial thresholds for features in various subsets highlights the importance of closely monitoring the deletion of features to avoid substantial deterioration in performance. The characteristics selected for each subgroup are listed in Table 3.

**Table 3:** Selected features for each subset.

Subsets	Selected features
Subset 1	‘dinpkt’, ‘sload’, ‘ct_dst_sport_ltm’, ‘dbytes’, ‘ct_dst_src_ltm’, ‘dttl’, ‘proto’, ‘sbytes’, ‘sttl’, ‘ct_state_ttl’, ‘service’
Subset 2	‘ct_dst_src_ltm’, ‘ct_srv_dst’, ‘dbytes’, ‘dmean’, ‘service’, ‘proto’, ‘sttl’
Subset 3	‘dmean’, ‘sttl’, ‘sinpkt’, ‘smean’, ‘sloss’, ‘dloss’, ‘proto’, ‘ct_srv_dst’, ‘ct_dst_src_ltm’, ‘state’, ‘dload’
Subset 4	‘dttl’, ‘ct_srv_dst’, ‘dbytes’, ‘ct_dst_src_ltm’, ‘sttl’, ‘proto’
Subset5	‘proto’, ‘service’, ‘sbytes’, ‘dbytes’, ‘sttl’, ‘sloss’, ‘smean’, ‘ct_dst_src_ltm’, ‘ct_srv_dst’
Subset 6	‘proto’, ‘service’, ‘sttl’, ‘response_body_len’
Subset 7	‘proto’, ‘ct_dst_src_ltm’, ‘sbytes’, ‘ct_srv_dst’, ‘dbytes’, ‘dload’, ‘dttl’, ‘synack’
Subset 8	‘ct_src_dport_ltm’, ‘sbytes’, ‘ct_dst_src_ltm’, ‘ct_srv_dst’, ‘dbytes’, ‘sttl’, ‘spkts’, ‘smean’, ‘dur’
Subset 9	‘service’, ‘sttl’, ‘ct_srv_dst’

### 3.3. Aggregating frequent features

Following the identification of the most suitable features for each subset, we conducted a thorough analysis to determine the frequency of occurrence of each characteristic across these subsets, as illustrated in Table 4. The assessment involved the classification of the characteristics based on their frequency of occurrence in order to assess their relative importance. Our attention was directed toward characteristics that were present in at least three distinct subgroups. This methodology ensured that our attention was directed toward characteristics that consistently influenced forecasting results across a variety of subgroups.

**Table 4:** Frequency of feature occurrence.

Feature names	Feature frequency
sttl	8
proto	7
ct_dst_src_ltm	7
ct_srv_dst	7
dbytes	6
service	5
sbytes	4
dttl	3
smean	3
sloss	2
dload	2
dmean	2

A collection of features and their respective frequencies of occurrence across various subsets are presented in the table, labeled as Table 4. Our selection procedure was informed by this information, which prioritized characteristics that consistently occurred and made a substantial contribution to the forecasts in each subgroup.

The features can be divided into three categories: High-Frequency Features, Moderate-Frequency Features, and Low-Frequency Features. In fact, the features “sttl” (which appears eight times), “proto,” “ct_dst_src_ltm,” and “ct_srv_dst” (which each appear seven times) were identified as highly influential. Their frequent selection suggests that they have a consistent and substantial influence on the predictive performance of a variety of subsets. While “dbytes,” “service,” and “sbytes” were present between four and six times, this indicates that they had a moderate but still significant impact on the predictive capabilities of the model.

Features that were not used more than three times, including “dload,” “dmean,” and “sloss,” were deemed less influential and were generally excluded from the final feature set in order to improve model performance and reduce noise.

Table 5 illustrates the final set of features that were employed for classification in order to enhance detection rates. The following is a description of each feature.

**Table 5:** Final Selected Features [36].

The choosing features	Description
sttl	Source to destination time to live
proto	Transaction protocol
ct_dst_src_ltm	No of connections of the same source 1 and the destination 3 address in 100 connections according to the last time 26
ct_srv_dst	No. of connections that contain the same service (14) and destination address (3) in 100 connections according to the last time (26).
dbytes	Destination to source bytes
service	http, ftp, ssh, dns..,else (-)
sbytes	Source to destination bytes
dttl	Destination to source time to live
smean	Mean of the flow packet size transmitted by the src

### 3.4. Classification

The classification performance of the XGBoost model is analyzed in this section. The model is trained under two distinct scenarios: using all available features and concentrating exclusively on the most impactful features identified in Table 5. Tables 6 and 7 provide a comprehensive comparison of the results derived from our proposed model and the outcomes of the model with all features.

**Table 6:** Experimental results.

	Accuracy	Precision	Recall	F1-score
All features	0.7289	0.6205	0.4097	0.3929
Selected Features	0.8353	0.7708	0.6388	0.6512

**Table 7:** The performance metrics of the two models in a variety of classes.

Class	Metric	All Features	Proposed Model
Normal	Precision	0.9572	0.9369
	Recall	0.8608	**0.9206**
	F1-score	0.9065	**0.9287**
Generic	Precision	0.9952	**0.9972**
	Recall	0.9764	**0.981**
	F1-score	0.9857	**0.989**
Exploits	Precision	0.7033	0.6424
	Recall	0.544	**0.9108**
	F1-score	0.6135	**0.7534**
Fuzzers	Precision	0.6565	**0.7531**
	Recall	0.6725	**0.7447**
	F1-score	0.6644	**0.7489**
DoS	Precision	0.2441	**0.4377**
	Recall	0.8063	0.1627
	F1-score	0.3747	0.2372
Reconnaissance	Precision	0.6923	**0.9165**
	Recall	0.0029	**0.7528**
	F1-score	0.0057	**0.8266**
Analysis	Precision	0.6444	0.5806
	Recall	0.0483	**0.1091**
	F1-score	0.0899	**0.1837**
Backdoor	Precision	0.6	**0.8837**
	Recall	0.0115	**0.1322**
	F1-score	0.0225	**0.23**
Shellcode	Precision	0.2115	**0.7505**
	Recall	0.0971	**0.8448**
	F1-score	0.1331	**0.7949**
Worms	Precision	0.5	**0.8095**
	Recall	0.0769	**0.8293**
	F1-score	0.1333	**0.8193**

Table 6 displays the overall performance metrics of two models. The selected features significantly improve the model’s accuracy, increasing it from 0.7289 to 0.8353. This indicates that the feature selection approach greatly enhances the model’s ability to reliably categorize occurrences throughout the whole dataset.

Furthermore, the model’s precision improves from 0.6205 when using all features to 0.7708 when using only selected features. A greater precision value implies that the model, using the selected features, has a lower rate of false positives.

This suggests that the model’s predictions are more dependable overall. The utilization of selected features in the model leads to a substantial improvement in recall, rising from 0.4097 to 0.6388. This rise demonstrates that the process of selecting features helps the model to more precisely identify occurrences that are real positives in the entire dataset, hence reducing the occurrence of false negatives.

Furthermore, the F1-score, which quantifies the equilibrium between precision and recall, experiences a significant boost from 0.3929 to 0.6512 when the chosen characteristics are utilized. The substantial rise indicates that the model, which incorporates the chosen features, achieves a more advantageous balance between precision and recall, leading to an overall enhancement in classification performance.

Subsequent analyses will demonstrate the performance of these models on each specific attack type, providing a more detailed understanding of how the models perform in identifying a variety of attacks.

Table 7 displays the performance characteristics of the two models across different classes. The suggested model exhibits a marginal enhancement in F1-score and recall, indicating a more equitable performance. Both models, utilizing selected features, attain elevated precision and recall for the “Normal” class. Both models have exceptional performance in the “Generic” category, exhibiting their efficacy in identifying generic attacks, with just slight variations in metrics.

**Fig 4:**
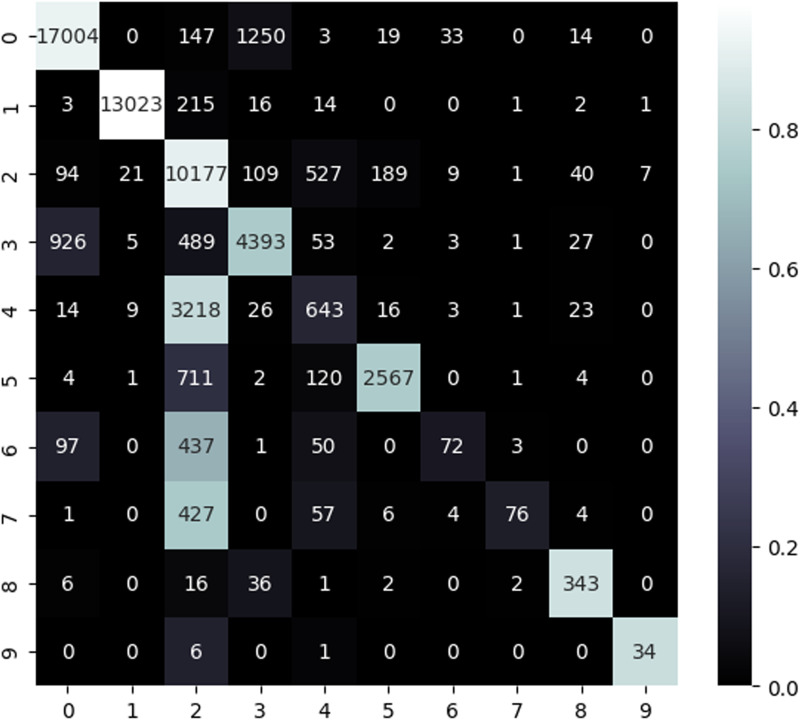
The confusion matrix of proposed model.

The proposed model for the “Exploits” class markedly improves the F1-score and recall, albeit it incurs a slight decrease in precision, signifying enhanced detection abilities overall. In the “Fuzzers” class, the suggested model attains superior precision and recall, resulting in more accurate detection of fuzzing attacks and an improved F1-score. The suggested approach enhances precision for the “DoS” class; however this results in a reduction in recall, indicating that certain DoS instances may be overlooked despite a decrease in false positives. Certain DoS records are misclassified as exploit assaults (Fig 4), perhaps due to feature overlap between the two groups. This problem is associated with the intrinsic data overlap in the UNSW-NB15 dataset, where common attributes among attack types hinder precise classification and lead to inaccuracies.

The suggested model exhibits a significant enhancement in F1-score and recall for the “Reconnaissance” class, facilitating improved detection of these hitherto underrepresented attacks when utilizing all features.

Classes including “Analysis,” “Backdoor,” “Shellcode,” and “Worms” demonstrate significant enhancements in precision, recall, and F1-score with the suggested model. These findings highlight the model’s superior capacity to detect infrequent or intricate attack types, which frequently pose difficulties for conventional classification methods.

**Fig 5:**
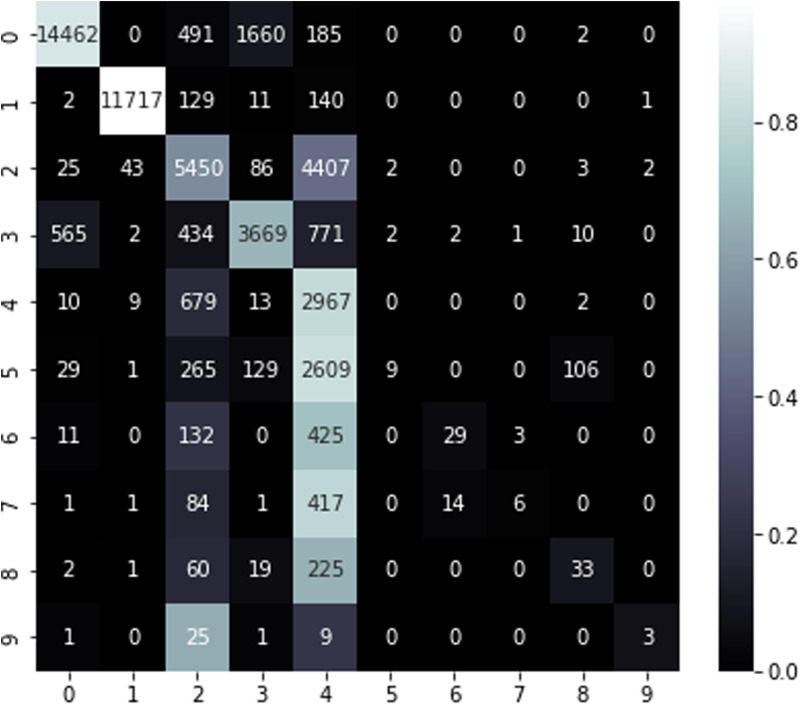
The confusion matrix using all features.

The confusion matrix for the model utilizing all attributes (Fig 5) reveals a substantial quantity of accurately classified instances for less complex classes such as “Normal” and “Generic” (diagonal components). Nevertheless, intricate categories such as “Exploits,” “Fuzzers,” and “DoS” demonstrate considerable misclassifications (off-diagonal elements). For example, whereas “DoS” cases attain high recall, the low precision underscores a predominance of false positives.

The confusion matrix for the proposed model utilizing selected characteristics (Fig 4) demonstrates improved classification performance across several classes. Accurate classifications are predominantly aligned along the diagonal, indicating a decrease in errors. The precision and recall metrics for “Reconnaissance,” “Shellcode,” “Backdoor,” “Generic,” and “Worms” reflect substantial enhancements, showcasing the proposed model’s enhanced efficacy in a multi-class context.

### 3.5. Validation of the proposed approach

To authenticate the suggested model, we used two contemporary and pertinent datasets: CICIDS2019 [37] and CICIoT2023 [38]. These datasets offer extensive assault scenarios and rectify the deficiencies identified in the UNSW-NB15 dataset. CICIoT2023 is notably important for include DoS assaults, which are susceptible to misclassification in the UNSW-NB15 dataset, facilitating a comprehensive assessment of the model across a wider spectrum of attack types.

The CICIDS2019 dataset, esteemed for its depiction of minority classes and modern attack tactics, was employed to validate our approach. It encompasses DDoS attacks that exploit application- and transport-layer protocols (e.g., UDP, TCP, or their mixes). Our assessment concentrated on the Day-1 subset, consisting of 88 characteristics and 569,650 records categorized as follows: Syn (352,968 instances), Portmap (42,606), LDAP (41,801), UDP (33,695), MSSQL (25,280), NetBIOS (16,252), UDPLag (83), and normal traffic (56,965). The existence of minority classes, including UDPLag, NetBIOS, and MSSQL, added complexity, underscoring the model’s capacity to identify infrequent and difficult attack types.

The CICIoT2023 dataset, released in 2023 [38], is derived from actual IoT devices and comprises 46,686,579 instances across 46 characteristics. It encompasses eight primary categories: one for standard traffic and seven for distinct attack types, namely DDoS, DoS, Reconnaissance, Web-based, Brute Force, Spoofing, and Mirai. A big difficulty is the class imbalance, with Web-based and Brute Force assaults being markedly underrepresented in comparison to DoS and DDoS categories. This disparity offered a chance to evaluate the model’s resilience in managing skewed data distributions.

Evaluating the model on these varied and unbalanced datasets confirmed its versatility across distinct assault scenarios. Table 8 encapsulates the findings, emphasizing the model’s efficacy across multiple categories.

**Table 8:** Performance evaluation of the proposed model across various classes in the CICIDS2019 and CICIoT2023 datasets.

Dataset	Class	Precision	Recall	F1-score
CICIDS2019	Normal	0.9993	0.9998	0.9998
	Syn	0.9989	0.9978	0.9979
	Portmap	0.9387	0.9543	0.9389
	LDAP	0.9380	0.9289	0.9412
	UDP	0.9900	0.9792	0.9858
	MSSQL	0.9521	0.9789	0.9683
	NetBIOS	0.9256	0.9489	0.9654
	UDPLag	0.7623	0.7745	0.7965
CIC IoT 2023	Normal	0.8791	0.9418	0.9094
	DDoS	0.9997	0.9998	0.9997
	DoS	0.9998	0.9989	0.9994
	Mirai	0.9997	1.0000	0.9999
	Spoofing	0.8638	0.8050	0.8333
	Recon	0.8921	0.9070	0.9134
	BruteForce	0.8471	0.7558	0.7989
	Web-based	0.8167	0.7313	0.7717

The findings illustrate the model’s outstanding performance across both datasets. In CICIDS2019, the model attained nearly flawless precision, recall, and F1-scores in classes such as Normal and Syn, while proficiently managing intricate attacks such NetBIOS (F1-score: 0.9654), MSSQL (0.9683), and LDAP (0.9412). The model notably detected minority classes, such as UDPLag, achieving an F1-score of 0.7965, highlighting its capacity to detect infrequent attack types.

At CICIoT2023, the model demonstrated exceptional proficiency in identifying DoS, DDoS, and Mirai assaults, with practically flawless scores. This is especially important for DoS, which previously encountered difficulties due to overlapping characteristics in other datasets. The explicit definition of DoS in CICIoT2023 facilitated the model’s exceptional performance. The model excelled at recognizing underrepresented assaults, specifically Web-based and Brute Force, achieving F1-scores of 0.7717 and 0.7989, respectively, so underscoring its efficacy in managing imbalanced data.

The model’s proficiency in accurately classifying both majority and minority groups enhances its relevance to real-world cybersecurity issues. These results confirm the model’s effectiveness in varied, intricate, and unbalanced settings, supporting conclusions drawn from the UNSW-NB15 dataset.

### 3.6. Comparison

The proposed approach, integrating XGBoost with ShapRFECV, exhibits enhanced efficacy relative to numerous current models across various datasets (Table 9). On UNSW-NB15, it attains an accuracy of 83.53%, marginally inferior to the Decision Tree’s 84.8%, although it substantially surpasses it in precision (77.08% compared to 63.3%), signifying a notable decrease in false positives. The suggested technique for CICIDS2019 achieves a state-of-the-art accuracy of 99.25%, exceeding competitors in precision (93.81%) and F1-score (94.92%), hence demonstrating its balanced and dependable performance. At CICIoT2023, it yields results that are equivalent to or superior to the Deep Neural Network (DNN) methodology, attaining an accuracy of 99.2% and an F1-score of 93.55%, significantly surpassing DNN’s recall (93.15% compared to 67.94%). Unlike models such as Logistic Regression, which depend on Principal Component Analysis and exhibit diminished precision and recall, or LSTM on NSL-KDD, which advantages from a less complex dataset, the proposed method consistently maintains equilibrium in performance metrics across intricate and imbalanced datasets, demonstrating its robustness and efficacy.

**Table 9:** Comparative analysis of results with existing models.

Model	Feature selection techniques	Dataset	Accuracy%	Precision%	Recall%	F1-score%
Logistic regression [39]	Principal component analysis &SMOTE	UNSW-NB15	68.2	57.8	58	54.8
Decision tree [33]	Correlation attribute	UNSW-NB15	84.8	63.3	60.9	61.9
SVM [40]	XGBoost	UNSW-NB15	–	53.95	61.52	51.31
ANN [40]	XGBoost	UNSW-NB15	77.51	–	–	–
DNN [41]	DT	–	82.1	–	–	–
LSTM [42]	Shap Value	NSL-KDD	99.74	95.42	94.42	94.9
DNN [38]	–	CICIoT2023	99,11	90,66	67,94	69,72
Proposed Approach: XGBoost	ShapRFECV	UNSW-NB15	83.53	77.08	63.88	65.12
		CICIDS 2019	99.25	93.81	94.53	94.92
		CICIoT2023	99.2	93.7	93.15	93.55

## 4. Conclusion

This paper introduces a novel methodology that combines the XGBoost model with SHAP-based Recursive Feature Elimination with Cross-Validation (ShapRFECV) to improve network intrusion detection on the UNSW-NB15 dataset, specifically targeting minority class intrusions. The model exhibited its efficacy by attaining a precision of 77.08% and an accuracy of 83.53%, highlighting its capacity to detect positive cases while reducing false positives. Its robustness and capacity for generalization were demonstrated by its outstanding identification of unusual and intricate assaults, such as Reconnaissance, Backdoor, Shellcode, and Worms, which are generally underrepresented in the dataset.

To enhance the model’s adaptability and reliability, we assessed it using the CICIDS2019 and CICIoT2023 datasets, which include more recent and varied attack scenarios. The tests validated the model’s capacity to generalize effectively and excel in practical intrusion detection scenarios.

Notwithstanding its advantages, the system possesses limits. A notable problem is the prolonged execution duration during the feature selection process, especially when retraining the model on extensive datasets or novel attack types. ShapRFECV is resource-intensive, potentially impeding the model’s efficiency, particularly in dynamic settings. We intend to investigate expedited feature selection methodologies, including ensemble approaches and genetic algorithms, to minimize processing duration while preserving superior feature selection quality.

A further drawback stems from the challenges in identifying specific attacks due to data overlap or shifts, as noted in the UNSW-NB15 dataset. Overlapping attributes between DoS and other attack categories might result in misunderstanding and misdiagnosis. To address this, we suggest the integration of concept drift detection methods, allowing the model to adjust to changing data and emerging assault patterns, thus enhancing accuracy amidst overlaps.

Additionally, to improve the model’s performance, we suggest utilizing sophisticated hyperparameter optimization methods, such as Bayesian optimization, for effective and focused exploration of the hyperparameter space. This method may optimize the model’s parameters and enhance its overall precision. Moreover, mitigating class imbalance by synthetic data generation employing Generative Adversarial Networks (GANs) may enhance the identification of underrepresented attacks by producing more equitable and authentic datasets. These enhancements would significantly reinforce the model’s resilience and efficacy in intrusion detection.

## References

[1] Eddermoug N, Mansour A, Sadik M, Sabir E, Azmi M. Klm-PPSA: Klm-based profiling and preventing security attacks for cloud environments: Invited Paper. 2019 International Conference on Wireless Networks and Mobile Communications (WINCOM). 2019. doi: 10.1109/wincom47513.2019.8942509

[2] N. 2., Eddermoug A, Mansour M, Sadik E, Sabir M, Azmi M. KLMbased profiling and preventing security attacks for cloud computing: a comparative study. Proceedings of the 28th International Conference on Telecommunications (ICT). 2021. p. 1–6.

[3] Abusitta A, Bellaiche M, Dagenais M. A trust-based game theoretical model for cooperative intrusion detection in multi-cloud environments. Proceedings of the 21st Conference on Innovations in Clouds, Internet and Networking Workshops (ICIN). 2018. p. 1–8.

[4] Resende PAA, Drummond AC. A Survey of random forest based methods for intrusion detection systems. ACM Comput Surv. 2018;51(3):1–36. doi: 10.1145/3178582

[5] Imperva. Bad Bot Report 2024; 2024. Retrieved from: https://palai.media/wp-content/uploads/2024/04/imperva-bad-bot-report-2024.pdf.

[6] Aburomman AA, Reaz MBI. A survey of intrusion detection systems based on ensemble and hybrid classifiers. Comput Secur. 2017;65:135–52. doi: 10.1016/j.cose.2016.11.004

[7] Bridges RA, Glass-Vanderlan TR, Iannacone MD, Vincent MS, Chen Q (Guenevere). A survey of intrusion detection systems leveraging host data. ACM Comput Surv. 2019;52(6):1–35. doi: 10.1145/3344382

[8] Sultana N, Chilamkurti N, Peng W, Alhadad R. Survey on SDN based network intrusion detection system using machine learning approaches. Peer-to-Peer Netw Appl. 2018;12(2):493–501. doi: 10.1007/s12083-017-0630-0

[9] Umer MF, Sher YB. Flow-based intrusion detection: techniques and challenges. Comput Secur. 2017-;70 238–54.

[10] Ren H, Li H, Liu D, Xu G, Cheng N, Shen XS. Privacy-preserving Efficient Verifiable Deep Packet Inspection for Cloud-Assisted Middlebox. IEEE Transactions on Cloud Computing; 2020.

[11] Aldweesh A, Derhab A, Emam AZ. Deep learning approaches for anomaly based intrusion detection systems: a survey, taxonomy, and open issues. Knowledge-Based Systems. 2020;189:105124. doi: 10.1016/j.knosys.2020.105124

[12] Masdari M, Khezri H. A survey and taxonomy of the fuzzy signature-based intrusion detection systems. Applied Soft Computing. 2020;106301.

[13] Masdari M, Khezri H. Towards fuzzy anomaly detection-based security: a comprehensive review. Fuzzy Optim Decis Making. 2020;20(1):1–49. doi: 10.1007/s10700-020-09332-x

[14] Pan Z, Hariri S, Pacheco J. Context aware intrusion detection for building automation systems. Computers & Security. 2019;85:181–201. doi: 10.1016/j.cose.2019.04.011

[15] Nisioti A, Mylonas A, Yoo PD, Katos V. From intrusion detection to attacker attribution: a comprehensive survey of unsupervised methods. IEEE Commun Surv Tutorials. 2018;20(4):3369–88. doi: 10.1109/comst.2018.2854724

[16] Liu L, Wang P, Lin J, Liu L. Intrusion detection of imbalanced network traffic based on machine learning and deep learning. IEEE Access. 2021;9:7550–63. doi: 10.1109/access.2020.3048198

[17] Bedi P, Gupta N, Jindal V. Siam-IDS: Handling class imbalance problem in Intrusion Detection Systems using Siamese Neural Network. Procedia Computer Science. 2020;171:780–9. doi: 10.1016/j.procs.2020.04.085

[18] Alabrah A. A Novel Study: GAN-Based Minority Class Balancing and Machine-Learning-Based Network Intruder Detection Using Chi-Square Feature Selection. Appl Sci. 2022;12(22):11662. doi: 10.3390/app122211662

[19] Elghalhoud O, Naik S, Zaman M, RicardoManzano S. Data balancing and CNN based network intrusion detection system. IEEE Wireless Communications and Networking Conference (WCNC). 2023. p. 1–6.

[20] Kasongo SM. An Advanced intrusion detection system for IIoT based on GA and tree based algorithms. IEEE Access. 2021;9:113199–212. doi: 10.1109/access.2021.3104113

[21] Gao X, Shan C, Hu C, Niu Z, Liu Z. An adaptive ensemble machine learning model for intrusion detection. IEEE Access. 2019;7:82512–21. doi: 10.1109/access.2019.2923640

[22] Kasongo SM, Sun Y. A deep learning method with wrapper based feature extraction for wireless intrusion detection system. Comput Secur. 2020;92:101752. doi: 10.1016/j.cose.2020.101752

[23] Anthi E, Williams L, Slowinska M, Theodorakopoulos G, Burnap P. A Supervised Intrusion Detection System for Smart Home IoT Devices. IEEE Internet Things J. 2019;6(5):9042–53. doi: 10.1109/jiot.2019.2926365

[24] Pham NT, Foo E, Suriadi S, Jeffrey H, Lahza HFM, Abramson D. Improving performance of intrusion detection system using ensemble methods and feature selection. Proceedings of Australian Computer Science Week (ACSW); 1–6.

[25] Devikrishna KS, Ramakrishna BB. An artificial neural network based intrusion detection system and classification of attacks. International Journal of Engineering Research and Applications (IJERA). 2013;3(4):1959–64.

[26] Kim G, Lee S, Kim S. A novel hybrid intrusion detection method integrating anomaly detection with misuse detection. Expert Systems with Applications. 2014;41(4):1690–700. doi: 10.1016/j.eswa.2013.08.066

[27] K. Maseer Z, Yusof R, A. Mostafa S, Bahaman N, Musa O, Ali Saleh Al-rimy B. DeepIoT.IDS: Hybrid Deep Learning for Enhancing IoT Network Intrusion Detection. Computers, Materials & Continua. 2021;69(3):3945–66. doi: 10.32604/cmc.2021.016074

[28] Guezzaz A, Benkirane S, Azrour M, Khurram S. A reliable network intrusion detection approach using decision tree with enhanced data quality. Security and Communication Networks. 2021;2021(1):1230593. doi: 10.1002/scn.1230593

[29] Batchu RK, Seetha H. An integrated approach explaining the detection of distributed denial of service attacks. Computer Networks. 2022;216 109269. doi: 10.1016/j.comnet.2022.109269

[30] Sharma NV, Yadav NS. An optimal intrusion detection system using recursive feature elimination and ensemble of classifiers. Microprocess Microsyst. [cited 2021 Sep 28]. 104293. Available from: https://scikit-learn.org/stable/modules/permutation_importance.html#relationto-impurity-based-importance-in-trees.

[31] Cloudstor. Unsw-nb15 csv files; 2020, [cited 2021 Jun 30] Available from: https://cloudstor.aarnet.edu.au/plus/index.php/s/2DhnLGDdEECo4ys?path=%2FUNSW-NB15%20-%20CSV%20Files

[32] Choudhary S, Kesswani N. Analysis of KDD-Cup’99, NSL-KDD and UNSW-NB15 Datasets using Deep Learning in IoT. Procedia Computer Science. 2020;167:1561–73. doi: 10.1016/j.procs.2020.03.367

[33] Zoghi Z, Serpen G. UNSW-NB15 computer security dataset: analysis through visualization. 2021.

[34] Moustafa N, Slay J. A hybrid feature selection for network intrusion detection systems: Central points. Proceedings of the 16th Australian Information Warfare Conference. 2015.

[35] Lundberg SM, Erion GG, Lee SI. Consistent individualized feature attribution for tree ensembles. arXiv preprint. 2018.

[36] Moustafa N, Slay J. UNSW-NB15: a comprehensive data set for network intrusion detection systems (UNSW-NB15 network data set). 2015 Military Communications and Information Systems Conference (MilCIS). 2015. p. 1–6. doi: 10.1109/milcis.2015.7348942

[37] Sharafaldin I, Lashkari AH, Hakak S, Ghorbani AA. Developing realistic distributed denial of service (DDoS) attack dataset and taxonomy. 2019 International Carnahan Conference on Security Technology (ICCST). 2019. p. 1–8.

[38] Neto ECP, Dadkhah S, Ferreira R, Zohourian A, Lu R, Ghorbani AA. CICIoT2023: A Real-Time Dataset and Benchmark for Large-Scale Attacks in IoT Environment. Sensors (Basel). 2023;23(13):5941. doi: 10.3390/s23135941 37447792 PMC10346235

[39] Ahmed HA, Hameed A, Bawany NZ. Network intrusion detection using oversampling technique and machine learning algorithms. PeerJ Computer Science. 2022;8:e820. doi: 10.7717/peerj-cs.820 PMC8771780 35111914

[40] Kasongo SM, Sun Y. Performance Analysis of Intrusion Detection Systems Using a Feature Selection Method on the UNSW-NB15 Dataset. J Big Data. 2020;7(1):. doi: 10.1186/s40537-020-00379-6

[41] AD E, Gao Q, Zhu M-Y, Chen Z, Na L. Network anomaly detection technology based on deep learning. 2021 IEEE 3rd International Conference on Frontiers Technology of Information and Computer (ICFTIC). 2021. p. 6–9.

[42] Asry CE, Benchaji I, Douzi S, Ouahidi BE. Effective Approaches for Intrusion Detection Systems in the Face of Low-Frequency Attacks. JAIT. 2024;15(9):1070–8. doi: 10.12720/jait.15.9.1070-1078

